# A Phylogenomic Perspective on the Radiation of Ray-Finned Fishes Based upon Targeted Sequencing of Ultraconserved Elements (UCEs)

**DOI:** 10.1371/journal.pone.0065923

**Published:** 2013-06-18

**Authors:** Brant C. Faircloth, Laurie Sorenson, Francesco Santini, Michael E. Alfaro

**Affiliations:** Department of Ecology and Evolutionary Biology, University of California Los Angeles, Los Angeles, California, United States of America; Field Museum of Natural History, United States of America

## Abstract

Ray-finned fishes constitute the dominant radiation of vertebrates with over 32,000 species. Although molecular phylogenetics has begun to disentangle major evolutionary relationships within this vast section of the Tree of Life, there is no widely available approach for efficiently collecting phylogenomic data within fishes, leaving much of the enormous potential of massively parallel sequencing technologies for resolving major radiations in ray-finned fishes unrealized. Here, we provide a genomic perspective on longstanding questions regarding the diversification of major groups of ray-finned fishes through targeted enrichment of ultraconserved nuclear DNA elements (UCEs) and their flanking sequence. Our workflow efficiently and economically generates data sets that are orders of magnitude larger than those produced by traditional approaches and is well-suited to working with museum specimens. Analysis of the UCE data set recovers a well-supported phylogeny at both shallow and deep time-scales that supports a monophyletic relationship between *Amia* and *Lepisosteus* (Holostei) and reveals elopomorphs and then osteoglossomorphs to be the earliest diverging teleost lineages. Our approach additionally reveals that sequence capture of UCE regions and their flanking sequence offers enormous potential for resolving phylogenetic relationships within ray-finned fishes.

## Introduction

The ray-finned fishes (Actinopterygii) constitute the dominant radiation of vertebrates on the planet including more than 32,000 species and equaling or exceeding richness estimates for the combined total of birds, mammals, and reptiles. Despite a long history of systematic study, resolution of phylogenetic relationships within this vast radiation remains an area of active research. Studies based upon traditional morphological and single-gene, PCR-based molecular approaches have succeeded in delineating several major lineages of ray-finned fishes, but conflict over how these lineages are related to one another remains. For example, the earliest morphological studies of ray-finned fishes unite gar (*Lepisosteus*) with the bowfin (*Amia*) in the clade Holostei [1] though this clade is not recovered in some later analyses [2], [3]. The early branching of teleost lineages has also been historically contentious. Systematists agree on the four earliest-diverging lineages: the osteoglossomorphs (bony-tongues; arawanas, elephant fishes, and allies), the elopomorphs (tarpons, bonefishes, and eels), the ostarioclupeomorphs (anchovies and herrings, minnows, characins, catfishes, and electric eels), and the euteleosts (salmons, pikes, lizardfishes, and perch-like fishes). However, there is disagreement over both the relationships among these groups and the basal divergences within euteleosts. Recent morphological and molecular studies have produced conflicting hypotheses of relationships among these lineages [4], [5], [7], [14]. Morphological analyses alternatively place the osteoglossomorphs [6] or the elopomorphs [7]–[10] as the sister group to all other teleosts and the remaining lineages sister to the ostarioclupeomorph/euteleost clade. Some molecular analyses place elopomorphs and osteoglossomorphs as the sister group to remaining teleosts [11], [12] while others recover a basal divergence between osteoglossomorphs and other teleosts [5], [13].

Recently, Near *et al.* [14] used wide-spread taxonomic sampling, in conjunction with sequence collected from nine commonly used nuclear genes, to provide a more comprehensive phylogenetic hypothesis of relationships among fishes. Their results supported the monophyly of the Holostei, suggesting that the elopomorphs formed the earliest diverging teleost lineage [14], and provided a new timescale for the divergence of ray-finned fishes. Although promising, these new insights into the radiation of actinopteryigians relied upon a relatively modest number of genomic markers, and the stability and timing of these relationships encoded throughout the genomes of the target groups remain largely untested. One exception to this statement includes a recent study by Zou *et al.* [15] that used transcriptome sequences to examine basal divergences within euteleosts. However, the Zou *et al.* [15] study did not include several anciently diverging lineages (e.g. *Amia*, osteoglossomorphs) informing questions about the early evolution of major groups of ray-finned fishes.

Phylogenomics and next-generation sequencing technologies offer enormous promise for resolving relationships within actinopterygians and other major sections of the Tree of Life. However, revolutions within genomics and informatics have had a surprisingly modest effect on data collection practices within the phylogenetics community: most studies of non-model organisms continue to rely upon direct sequencing of a moderate number of loci, and workflows that do take advantage of massively parallel sequencing platforms remain bottlenecked by cross-species amplification of phylogenetically informative loci. Several alternatives to traditional phylogenetic workflows exist that help to overcome the inefficiencies of gene-based sequencing. One class of these methods is exemplified by the recent work of Zou *et al.* [15], who used a combination of *de novo* transcriptome sequencing, existing transcript data, and computational methods to identify 274 orthologous groups from which they inferred the phylogeny of the Actinopterygii. The benefits of their approach include the use of existing, transcript-related data sets (ESTs in GenBank); reasonably well-established data generation methods; and the collection of data from hundreds of loci across the genomes of the focal taxa. Limitations of this approach include reliance on sampling fresh or properly preserved tissues (generally precluding the use of thousands of existing museum samples), dependence of the approach on expression patterns of the tissue sampled, and collection of data from fewer genomic locations than alternative methodologies.

A second class of phylogenomic methods involves sequence capture of nuclear regions flanking and including ultraconserved elements (UCEs) [16]. Rather than sequencing expressed portions of the genome, the UCE-based approach involves enriching organismal DNA libraries for hundreds to thousands of UCEs and their flanking regions; sequencing these libraries using massively parallel sequencing; and assembling, aligning, and analyzing the resulting data using informatic tools. This approach has been successfully used in mammals [17], birds [16], [18], and reptiles [19] to generate phylogenomic data sets that contain at least one order of magnitude more characters than those generated using PCR and to resolve historically contentious sections of the Tree of Life [17], [19]. The UCE approach differs from transcript-based phylogenomic studies [15] because data collection is independent of expression pattern, researchers can prepare and enrich libraries from existing tissue collections, and UCE loci may be better conserved and more numerous across distantly related taxa [17].

Here, we apply the UCE approach to ray-finned fishes by developing a novel set of sequence capture probes targeting almost 500 UCE regions in ray-finned fishes. We use the UCE data to provide the first phylogenomic perspective based upon widespread sampling of hundreds of markers across the genome on long-standing controversies regarding relationships at the base of the ray-finned fish Tree of Life. These include whether *Lepisosteus* and *Amia* form a monophyletic group (the Holostei [1], [20]) and how the major lineages of teleosts, which constitute >99% of ray-finned fishes, are related to one another [4], [5], [7]–[10], [21], [22]. Our results reveal that sequence capture of UCE regions can efficiently and economically generate massive data sets with strong resolving power at both deep and shallow phylogenetic scales within fishes.

## Results and Discussion

### Probe design, UCE enrichment, and sequencing

We located 500 UCEs shared among all actinopterygian fishes. The total number of UCEs we found in actinopterygians is smaller than in birds [16] and in mammals [17] which likely reflects both the greater phylogenetic depth spanned by fishes and the paucity of genome-enabled taxa allowing comparisons across this clade. We designed a set of 2,000 capture probes targeting each of these loci (4× tiling). Following enrichment and sequencing, we obtained an average of 2,819,047 reads per species, which we assembled into an average of 665 contigs having an average length of 457 bp (Table 1). After removing contigs that matched no UCEs and UCE loci that matched multiple contigs, we enriched an average of 332 unique contigs matching UCE loci from each species. Average sequencing depth across unique UCE loci was 498X. An average of 55% of assembled contigs (95% CI
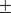
0.10; min
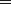
0.15; max
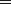
0.88) were on-target while an average of 32% of reads were on-target (95% CI
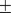
0.08; min
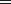
0.07; max
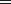
0.62). The variance in the proportion of reads and contigs on-target suggests that input DNA quality, insert length of DNA libraries, and taxonomic distance between the taxon used to design probes and taxa from which we enriched UCEs may play a role in enrichment efficiency. However, the lowest enrichment efficiencies we observed resulted from our removal of duplicated ultraconserved elements that may result from lineage-specific duplication events (e.g., *Salvelinus fontinalis* [23] prior to computing the proportion of reads and contigs on-target.

**Table 1:** Sequence read and assembly statistics for fish species used in this study.

Scientific name	Common name	Number of trimmed reads	Contigs assembled	Reads in contigs	UCE contigs	Reads in UCE contigs	Avg. size	Avg. coverage	Contigs on target	Reads on target
*Umbra limi*	central mudminnow	2,727,071	1109	740,079	409	564,715	508.8	267.4	0.37	0.21
*Diaphus theta*	California headlightfish	2,626,413	584	688,635	401	604,295	502.4	299.1	0.69	0.23
*Antennarius striatus*	striated frogfish	3,724,320	474	2,462,193	418	2,310,186	649.7	850.2	0.88	0.62
*Megalops* sp.	tarpon	2,771,805	786	650,577	247	231,314	485.4	191.5	0.31	0.08
*Astyanax fasciatus*	banded astyanax	2,731,668	543	1,444,767	355	1,211,903	526.2	657.2	0.65	0.44
*Acanthurus japonicus*	Japan surgeonfish	2,017,174	613	1,242,932	454	1,125,871	600.8	405.9	0.74	0.56
*Amia calva*	bowfin	2,619,643	562	1,608,614	366	1,368,091	578.9	646	0.65	0.52
*Lampris guttatus*	opah	2,472,439	486	1,350,852	418	1,237,650	568.7	520.2	0.86	0.50
*Acipenser fulvescens*	lake sturgeon	3,083,152	577	1,129,829	167	467,414	426.9	665.4	0.29	0.15
*Anchoa compressa*	deep body anchovy	2,617,717	533	783,323	287	625,862	448.6	479.2	0.54	0.24
*Danio rerio*	zebrafish	2,777,132	518	1,367,065	382	1,166,020	463.4	657.1	0.74	0.42
*Polypterus senegalus*	gray bichir	3,206,418	576	873,104	294	726,100	557.6	440	0.51	0.23
*Pantodon buchholzi*	freshwater butterflyfish	3,329,691	466	2,058,929	272	1,399,286	550.4	930.5	0.58	0.42
*Strophidon sathete*	slender giant moray	3,159,269	1007	448,390	277	246,758	510.6	172.9	0.28	0.08
*Osteoglossum bicirrhosum*	silver arawana	2,735,138	643	1,565,346	276	813,175	467	623.9	0.43	0.30
*Salvelinus fontinalis*	brook trout	2,466,696	1118	688,684	166	161,214	408.7	234.8	0.15	0.07
*Taenianotus triacanthus*	leaf scorpionfish	3,245,453	712	1,423,244	447	1,252,564	652.5	431.4	0.63	0.39

We integrated extant genomic data from several fish species to this group of unique UCE contigs, and we constructed 491 alignments (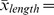
305 bp, 95% CI
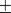
16.0) comprising 149,366 characters. After trimming alignment edges and removing taxa with excessively trimmed data, each alignment contained an average of 21 target taxa (95% CI
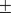
0.4; min
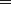
3 taxa; max
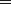
27 taxa). We removed two loci from further consideration because we were unable to estimate site-rate substitution models for these loci due to their short lengths. The resulting incomplete data matrix contained 489 loci (149,246 characters; 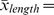
305 bp, 95% CI
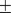
16.0). We used this incomplete data matrix for subsequent analyses with RAxML and MrBayes. After removing loci having missing data for *Polypterus* and *Acipenser*, we input 136 alignments (41,731 characters; 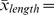
307 bp, 95% CI
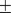
27.7) to CloudForest for model selection and subsequent species tree estimation using STAR.

### A phylogenomic perspective on the basal radiation of ray-finned fishes

**Figure 1:**
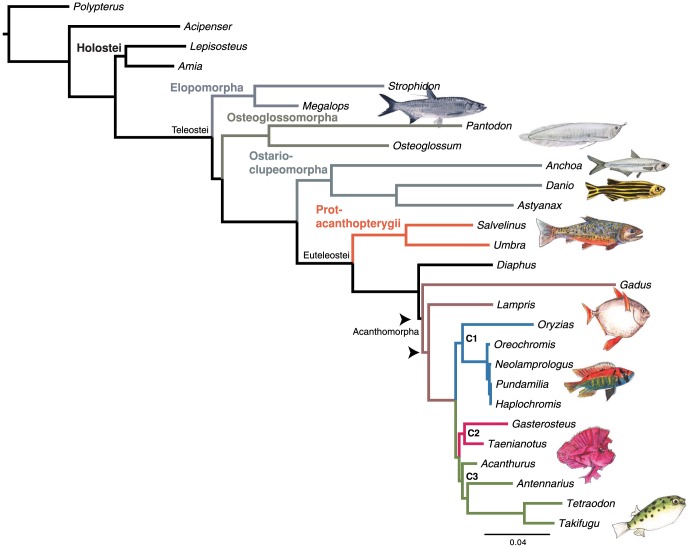
Maximum likelihood phylogram of ray-finned fish relationships based upon UCE sequences. All nodes except for two (indicated by arrows) supported by bootstrap proportions and Bayesian posterior probabilities 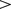
0.99. Our analysis supports a monophyletic Holostei and reveals the elopomorphs to be the earliest diverging lineage of teleosts. C1, C2, and C3 indicate clades within acanthomorphs consistent with other recent molecular studies (see Discussion).

Maximum likelihood analysis produced a single, completely resolved topology wherein all but two nodes received high (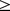
0.99) bootstrap proportions and Bayesian posterior probabilities (Fig. 1). This topology provides new insight into several long-standing questions concerning the evolution of ray-finned fishes. Our analysis strongly supports the monophyly of the Holostei (*Amia*+*Lepisoteus*). This clade is historically controversial because morphological studies alternatively support [1], [20] and refute [2], [3] the monophyly of this group, while recent molecular studies generally recover the relationship [14], [24], [25]. Additionally, our analyses do not support prior findings of an “ancient fish clade” including the Holostei+Acipenseriformes as the sister group to the teleosts [25], [26]. Rather, our results strongly suggest a traditional relationship in which these lineages form successive sister groups to the teleosts.

Our phylogenomic data provide strong evidence for the placement of elopomorphs as the sister group to all other teleosts and osteoglossomorphs and ostarioclupeomorphs as successive sister lineages to the euteleosts (Fig. 1). Our maximum likelihood topology is strongly incongruent with mitogenomic studies [5], [13] but consistent with both a recent analysis of multiple nuclear genes [14] and some of the earliest morphological analyses of the group [7]–[10]. Within euteleosts, our results are congruent with recent molecular studies [4], [14], [15] in placing esociforms as the sister to salmoniforms rather than any neoteleost lineages.

Within acanthomorphs, the largest clade of euteleosts, UCEs recover several intriguing clades that agree with results from recent molecular phylogenetic studies. These include the African cichlids+medaka (Clade C1, Fig. 1), corresponding to an expanded clade of atherinomorphs suggested by recent studies [15], [27], [28]; a clade of gasterosteiforms (stickleback) and scorpaeniforms (*Taenionotus*) that is congruent with recent molecular and morphological studies [15], [29], [30]; and a clade including surgeonfish, frogfishes, and pufferfishes (acanthuroids, lophiiforms, and tetraodontiforms) corresponding to acanthomorph clade “N” of Dettai and Lecointre [14], [31]. Based upon previous time-calibrated studies [14], [32] and preliminary divergence time analyses of the UCE data set [33], our results suggest that UCEs provide sufficient phylogenetic signal to resolve splits within haplochromine cichlids that may be less than 5 Ma old [32] as well as the most basal actinopterygian divergences that exceed 400 Ma.

The STAR topology was less resolved than topologies based upon analyses of the concatenated data set (Fig. S1) but recovered largely congruent relationships including a monophyletic Holostei as the sister to other actinopterygians; monophyly of elopomorphs, osteoglossomorphs, ostarioclupeomorphs, and euteleosts; and a successive sister group relationship between ostarioclupeomorphs, *Salvelinus*+*Umbra*, and all remaining euteleosts. The species tree switched the position of the Gadiformes, represented by cod (*Gadus*) and Myctophiformes, represented by *Diaphus*. This position is not congruent with results from Near *et al.* [14] but has been suggested by previous molecular studies [4], [24], [34]. Relationships within cichlids are not fully resolved, but we recovered strong support for a clade consisting of *Neolamprologus*, *Haplochromis*, and *Oreochromis* that is not congruent with the concatenated topology (Fig. 1) or with accepted cichlid relationships [35].

Although UCE data would seem to provide a good fit to gene-tree species tree approaches because of the large number of loci that the approach generates, there are several challenges that genomic scale empirical data sets pose to accurate species tree reconstruction. These include pervasive incomplete taxonomic sampling across UCE loci and insufficient resolution of individual gene trees due to the recovery of relatively short contigs. Further refinement of the protocols developed here, including modification of the in vitro transposition reaction to yield longer insert lengths; replacement of transposase-mediated library preparation with physical shearing by sonication and T/A ligation; size-selection of enriched, amplified libraries; deeper sequencing of longer libraries; paired-end reads; and longer sequence read lengths should improve gene-tree species tree reconstruction by increasing the amount of flanking sequence recovered across individual UCEs. Additional optimization of probe-designs, tiling densities, hybridization conditions, and hybridization reactions should increase the proportion of UCE loci recovered across individual taxa.

### Conclusions

Sequence capture of regions anchored by UCEs offers a powerful and efficient means of generating massive genomic data sets capable of resolving phylogenetic relationships at both deep and shallow scales in non-model organisms. Our UCE-based approach offers several advantages over previous studies that should contribute to the reliability of our topology. These benefits include efficient sampling of sequence data across individual genomes and among divergent taxa, collection of data from an order of magnitude more loci than studies based upon traditionally used genetic markers and almost twice as many loci as transcriptome-based genomic studies [15], validity of the UCE probe set across bony fishes spanning 400 Ma of evolutionary history, and utility of the UCE enrichment approach with tissues collected from museum specimens. Additionally, these data illustrate that biologists can use UCE-based genetic markers to reconstruct the phylogeny of taxa other than amniotes, supporting the observation that UCE-based markers are a universal source of phylogenetically informative characters [16], [17].

### Availability

Contigs assembled from raw read data are available from NCBI Genbank (Accession #s: JQ717376–JQ723011). Probe data, assembled contigs, alignments, and data sets we used for analysis are available from Dryad (doi: 10.5061/dryad.j015n). Software used for the analysis of raw sequence data are available under an open-source, BSD license from https://github.com/faircloth-lab/phyluce, https://github.com/faircloth-lab/illumiprocessor, and https://github.com/ngcrawford/cloudforest. Protocols for library preparation and UCE enrichment are available under Creative Commons license from http://ultraconserved.org.

## Materials and Methods

### Ethics statement

All tissues used in this study were either received as loans from the Field Museum, Virginia Institute of Marine Science, or Scripps Institution of Oceanography or collected under Institutional Animal Care and Use Committee (IACUC) protocols #17611 (University of California, Los Angeles), #12790 (University of California, Davis), or #16956 (University of California, Davis).

### Identification of UCE regions

To identify ultraconserved elements (UCEs) in fishes, we used genome-to-genome alignments of stickleback (*Gasterosteus aculeatus*) to medaka (*Oryzias latipes*) to locate nuclear DNA regions of 100% conservation greater than 80 bp in length. To enable efficient capture-probe design, we buffered these regions to 180 bp (where needed) by including equal amounts of medaka sequence 5′ and 3′ to each UCE. We aligned or re-aligned these buffered regions to the genome-enabled fishes (zebrafish, *Danio rerio*, stickleback, medaka, and two species of puffers, *Tetraodon nigroviridis* and *Takifugu rubripes*) using LASTZ [36], keeping only non-duplicate matches of 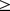
120 bp and 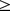
80% sequence identity across all species in the set. Based on the intersection of UCE loci across all fishes that were greater than 10 Kbp apart, we designed a pilot set of 120 bp sequence capture probes for each of the UCEs present among all members of the set by tiling probes at 4× density. We had these probes commercially synthesized into a custom SureSelect target enrichment kit (Agilent, Inc.). We used a higher than normal [37] tiling density to help ameliorate potential sequence differences among species introduced by buffering shorter UCEs to 180 bp.

### Library preparation, UCE enrichment, sequencing, and assembly

Tissues used in this study were received as loans with permission from the Field Museum, Virginia Institute of Marine Science, or Scripps Institution of Oceanography or collected under IACUC protocols #17611, #12790, and #16956.

We extracted DNA from tissues using phenol-chloroform techniques or DNEasy kits (Qiagen Inc.), treated extracts with RNase, and followed RNase treatment with column-based cleanup (Qiagen Inc.). We prepared DNA libraries from 18 fish species, including representatives of five acanthomorph orders and two families of perciforms (Table 1), by slightly modifying the Nextera (Epicentre Biotechnologies) library preparation protocol for solution-based target enrichment [16] and increasing the number of PCR cycles following the tagmentation reaction to 20. The Nextera library preparation protocol uses in vitro transposition followed by PCR to shear DNA and attach indexed sequencing adapters [38] rather than relying on physical shearing followed by standard T/A ligation. Transposase-mediated library preparation using the Epicentre Nextera kit produces libraries with insert sizes averaging 100 bp (95% CI: 45 bp) [38]. Following library preparation, we substituted a blocking mix of 500 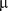
M (each) oligos composed of the forward and reverse complements of the Nextera adapters for the Agilent-provided adapter blocking mix (Block #3). We incubated species-specific libraries (500 ng) with synthetic RNA probes from the SureSelect kit for 24 h at 65

C. We followed the standard SureSelect protocol to enrich DNA libraries following hybridization; we eluted clean, enriched DNA in 30 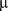
L of nuclease free water; and we used 15 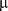
L of enriched template in a 50 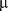
L PCR reaction of 20 cycles combining forward, reverse, and indexing primers with Nextera polymerase to add a custom set of 24 indexed adapters [39]. We cleaned PCR reactions using Agencourt AMPure XP. We quantified enriched, indexed libraries using qPCR (Kapa Biosystems), and we prepared two library pools containing 10 libraries at equimolar ratios prior to sequencing.

We sequenced each pool of enriched DNA using two lanes of a single-end 100 bp Illumina Genome Analyser (GAIIx) run. After sequencing, we trimmed adapter contamination, low quality bases, and sequences containing ambiguous base calls using a pipeline we constructed (https://github.com/faircloth-lab/illumiprocessor). We assembled reads, on a species-by-species basis, into contigs using Velvet [40] and VelvetOptimiser (https://github.com/Victorian-Bioinformatics-Consortium/VelvetOptimiser). Following assembly, we used a software package (https://github.com/faircloth-lab/phyluce) containing a custom Python program (match_contigs_to_probes.py) integrating LASTZ [36] to align species-specific contigs to the set of probes/UCEs we used for enrichment while removing reciprocal and non-reciprocal duplicate hits from the data set. During matching, this program creates a relational database of matches to UCE loci by taxon. This program also has the ability to include UCE loci drawn from existing genome sequences, for the primary purpose of including available data from genome-enabled taxa as outgroups or to extend taxonomic sampling. We used this feature to include UCE loci we identified in the genome sequences of *Gasterosteus aculeatus*, *Haplochromis burtoni*, *Neolamprologus brichardi*, *Oreochromis niloticus*, *Oryzias latipes*, *Pundamilia nyererei*, *Takifugu rubripes*, *Tetraodon nigroviridis*, *Gadus morhua*, and *Lepisosteus oculatus*. After generating the relational database of matches to enriched sequences and genome-enabled taxa, we used additional components of PHYLUCE (get_match_counts.py) to query the database and generate fasta files for the UCE loci we identified across all taxa. Then, we used a custom Python program (seqcap_align_2.py) to align contigs with MAFFT [41] and trim contigs representing UCEs, in parallel, across the selected taxa prior to phylogenetic analysis [16].

### Phylogenetic Analyses

The large number of UCE loci we collected create a vast potential space for partitioning data that makes a traditional evaluation of alternative partitioning strategies computationally challenging. As a result, we modeled nucleotide substitutions across the concatenated data set using two approaches. For Bayesian analysis, we used a custom script (run_mraic.py) wrapping a modified MrAIC 1.4.4 [42] to find the best-fitting, finite-sites substitution model for each UCE locus, we grouped loci having similar substitution models (selected by AICc) into the same partition, and we assigned the partition specific substitution model to all loci concatenated within each partition. For maximum likelihood analyses, we maintained the partitions identified in the Bayesian analysis and we modeled each partition using the GTR+CAT approximation. We performed Bayesian analysis of the concatenated data set using MrBayes 3.1 [43] and two independent runs (4 chains each) of 5,000,000 iterations each, sampling trees every 500 iterations, to yield a total of 10,000 trees. We sampled the last 5,000 trees after checking results for convergence by visualizing the log of posterior probability within and between the independent runs for each analysis, ensuring the average standard deviation of split frequencies was 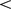
0.001, and ensuring the potential scale reduction factor for estimated parameters was approximately 1.0. We performed maximum likelihood analysis of the concatenated data in RAxML [44] using the rapid bootstrapping algorithm and 500 bootstrap replicates.

Gene tree-species tree methods enjoy some advantages over the analysis of concatenated data sets under certain conditions [45]–[47] but may also be sensitive to missing data [48] and to the resolution of individual gene trees [49]. To minimize the number of unresolved gene tree topologies and maximize the number of topologies that overlapped in sampling the base of the actinopterygian tree, we selected a subset of the UCE contigs containing complete data for *Polypterus* and *Acipenser* and loci 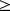
50 bp, and we used this subset to estimate a species tree with CloudForest (https://github.com/ngcrawford/CloudForest), a parallel implementation of a workflow combining substitution model selection (similar to MrAIC 1.4.4 [42]) and gene tree estimation using PhyML [50]. We estimated the species tree by summarizing gene trees using STAR [51]–[53]. To assess confidence in the resulting species tree, we used CloudForest to generate 1000, multi-locus, non-parametric bootstrap replicates by resampling nucleotides within loci as well as resampling loci within the data set [54], we summarized bootstrap replicates using STAR, and we reconciled bootstrap replicates with the species tree using RAxML.

## Supporting Information

Figure S1
**Species tree based upon STAR analysis.** Topology based upon analysis of all loci 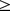
50 base pairs that contained both *Polypterus* and *Acipenser* (N = 136). Node values indicate bootstrap proportion based upon 1000 replicates. We collapsed nodes having 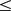
50% bootstrap support.(EPS)
